# Detection of 17 β-Estradiol in Environmental Samples and for Health Care Using a Single-Use, Cost-Effective Biosensor Based on Differential Pulse Voltammetry (DPV)

**DOI:** 10.3390/bios7020015

**Published:** 2017-03-29

**Authors:** Yifan Dai, Chung Chiun Liu

**Affiliations:** Department of Chemical & Biomolecular Engineering and Electronics Design Center, Case Western Reserve University, 10900 Euclid Avenue, Cleveland, OH 44106, USA; yxd176@case.edu

**Keywords:** 17 β-estradiol, estrogen pollution, female reproduction, DPV

## Abstract

Environmental estrogen pollution and estrogen effects on the female reproductive system are well recognized scientifically. Among the estrogens, 17 β-estradiol is a priority in environmental estrogen pollution, and it is also a major contributor to estrogen which regulates the female reproductive system. 17 β-estradiol is carcinogenic and has a tumor promotion effect relating to breast cancer, lung cancer and others. It also affects psychological well-being such as depression, fatigue and others. Thus, a simple method of detecting 17 β-estradiol will be important for both environmental estrogen pollution and health care. This study demonstrates a single-use, cost-effective 17 β-estradiol biosensor system which can be used for both environmental and health care applications. The bio-recognition mechanism is based on the influence of the redox couple, K_3_Fe(CN)_6_/K_4_Fe(CN)_6_ by the interaction between 17 β-estradiol antigen and its α-receptor (ER-α; α-estrogen antibody). The transduction mechanism is an electrochemical analytical technique, differential pulse voltammetry (DPV). The levels of 17 β-estradiol antigen studied were between 2.25 pg/mL and 2250 pg/mL; Phosphate buffered saline (PBS), tap water from the Cleveland regional water district, and simulated urine were used as the test media covering the potential application areas for 17 β-estradiol detection. An interference study by testosterone, which has a similar chemical structure and molecular weight as those of 17 β-estradiol, was carried out, and this 17 β-estradiol biosensor showed excellent specificity without any interference by similar chemicals.

## 1. Introduction

Estrogen is a steroid hormone which is directly responsible for the development and regulation of the female reproductive system. Furthermore, estrogen is considered to be carcinogenic and has a tumor promotion effect [1,2,3]. Its level related to the risk of breast cancer is evident [4,5,6,7]. In terms of human health, the estrogen level in women is also related to lung cancer [8], uterine (endometrial) and ovarian cancers [9,10], even though the exact mechanism of the cancer development is not entirely understood. It is also well recognized that, psychologically, the level of the estrogen in women can affect weight gain, depression, fatigue, mood swings, trouble sleeping and others. Consequently, an estrogen or estradiol test or physician-prescribed estrogen therapy may be helpful in addressing the impact of estrogen levels in women. 

Estrogen contamination in the environment due to the large quantity of natural estrogen from human urine disturbs the endocrine system in the ecosystem, and it is well recognized [11,12,13]. Pollution of the environment and food supply caused by estrogenic chemicals are well acknowledged. It is well documented that estrogen pollution causes the death and deformation of birds, fishes, animals as well as human beings [14,15]. Specifically, the Water Framework Directive (WFD) of the European Union listed 17 β-estradiol as a priority pollutant of estrogens [16,17]. Therefore, for both biomedical and environmental health reasons, the detection of estrogen is of scientific and health importance.

There are instrumental analysis techniques of measuring estrogen, including high-performance liquid chromatography (HPLC), gas chromatography/mass spectroscopy (GC/MS) and others [18]. These analyses are very sensitive and accurate, but are also very complicated to perform, requiring expensive instruments and well-trained operators. Consequently, a simpler and less expensive measurement technology of estrogen will be of scientific and commercial importance. Biosensors are one of the potential technologies which can minimize the shortcomings of the current detection technologies mentioned above, providing a simpler and sensitive detection method of estrogen. 

There are three major forms of natural estrogen: estrone, estradiol and estriol [19,20]. Estradiol is the most important one among these three major forms of estrogen, and 17 β-estradiol is of foremost importance in both medical and environmental estrogen assessment [21,22]. Thus, the development of a simple, cost-effective detection method of 17 β-estradiol will be meaningful and it is the objective of this study. Specifically, a cost-effective, single-use, in vitro or in situ 17 β-estradiol detection biosensor is developed for this practical application. This 17 β-estradiol biosensor is portable and simple to operate, and suitable for both health care and environmental applications. 

Biosensor uses for the measurement of 17 β-estradiol have been exploited by different groups of researchers [23,24]. These reported approaches have their own merits and limitations. In some cases, the sensitivity of the detection was limited. In other cases, the quantitation of nano-gold particles used for each single electrode element of the biosensor was difficult, making the practical applications of the estrogen biosensor impossible and expensive. The transduction mechanism of this study was differential pulse voltammetry (DPV), which required 30 s for a complete measurement; while others used electrochemical impedance spectroscopy (EIS) or AC impedance measurement would require 600 s or longer. Thus, our DPV measurement was much more time-efficient. Furthermore, our thin gold film-based electrode was prepared by sputtering physical vapor deposition, which was accomplished on an atomic level deposition, providing uniform and reproducible electrode surface and higher sensor sensitivity. In order to minimize the shortcomings in detecting 17 β-estradiol, a cost-effective, single-use, disposable biosensor for practical applications is undertaken in this research.

In this study, the bio-recognition mechanism of this biosensor was based on the influence of the redox coupling reaction, K_3_Fe(CN)_6_/K_4_Fe(CN)_6_ by the 17 β-estradiol antigen and its α-receptor (ER-α; α-estrogen antibody). Antibody and antigen interaction was a “lock-and-key” one-to-one combination providing the specificity of the biosensor. In the detection of 17 β-estradiol, the estrogen receptor α (ER-α; α-estrogen antibody) is used to provide this lock-and-key bio-recognition mechanism. This α-estrogen interacts with 17 β-estradiol affecting the electron charge transfer and can influence a redox coupling reaction in the test medium [17]. Consequently, the level of 17 β-estradiol can be assessed. Researchers have used different electrochemical analytical techniques, such as electrochemical impedance spectroscopy (EIS) square-wave voltammetry and others, as well as various electrode materials including glassy carbon, graphene and others for the detection of 17 β-estradiol [17,25,26,27,28,29,30]. 

The fabrication of the biosensor used in this study employed sputtering—a physical vapor deposition (PVD) technique—to formulate the thin-film gold working and counter electrode elements of the biosensor; it was deposited at an atomic level resulting in the very uniform and reproducible electrode elements. This fabrication step could be accomplished on a roll-to-roll manufacturing process and it was cost-effective. This biosensor had a three-electrode configuration, and the reference electrode was a thick-film printed Ag/AgCl electrode. Laser ablation technique was used to define the structure and size of the biosensor elements. 

Differential pulse voltammetry (DPV) of electrochemical analytical technique was employed as the transduction mechanism of this biosensor. DPV applied a linear sweep voltammetry with a series of regular voltage pulses superimposed on the linear potential sweep. The current was then measured immediately before each potential change. Thus, the effect of the charging current could be minimized, achieving a higher sensitivity. Furthermore, the K_3_Fe(CN)_6_/K_4_Fe(CN)_6_ redox coupling reaction was used, demonstrating the effect of 17 β-estradiol and α-estrogen antibody interaction in the test medium. It was based on the unique design and fabrication of the biosensor and the application of DPV measurement that this cost-effective, single-use, disposable in vitro and in situ biosensor for estrogen, specifically 17 β-estradiol, was successfully developed. Phosphate buffer saline (PBS), normal tap water (from the Cleveland regional water district) and simulated urine were used as the test media. These tests sustained that this biosensor could be used for both human care and environmental applications. 17 β-estradiol in the concentration range of 2.25–2250 pg/mL was used in this study, covering a wide range of 17 β-estradiol concentrations.

## 2. Materials and Methods

### 2.1. Apparatus and Reagents

Phosphate Buffer Solution (PBS) 1.0 M (pH 7.4) (Cat. #P3619), 3-Mercaptopropionic acid (MPA) (Cat. #5801), N-(3-dimethylaminopropyl)-N′-ethylcarbodiimide hydrochloride (EDC) (Cat. #E1769), and N–hydroxysuccinimide (NHS) (Cat. #130672) were purchased from Sigma-Aldrich (St. Louis, MO, USA). 17 β-estradiol (Cat. #E8875) was also obtained from Sigma-Aldrich (St. Louis, MO, USA) and anti-estrogen receptor, α-antibody [E-115] of estrogen (Cat. #ab32063) was purchased from ABCAM (Cambridge, MA, USA). Potassium hydroxide pellets (Cat. #P1767), concentrated H_2_SO_4_ 95.0 to 98.0 w/w % (Cat. #A300) and concentrated HNO_3_ 70 w/w % (Cat. #A200) were received from Fisher Scientific (Pittsburgh, PA, USA). Dimethyl sulfoxide (DMSO) (Cat. #BP231-1) was also obtained from Fisher Scientific (Pittsburgh, PA, USA). Simulated urine, normal (Cat. #695955) was purchased from the Carolina Biological Supply Co. (Burlington, NC, USA). For the interference study, testosterone C-111N (Cat. #T1500) from Sigma-Aldrich (St. Louis, MO, USA) was obtained. Testosterone was a controlled substance and required special permission to obtain the chemical. All the chemicals were used without further purification. A CHI 660C (CH Instrument, Inc., Austin, TX, USA) Electrochemical Workstation was used for DPV and EIS investigations. Similar Model CHI 660 A-E Electrochemical Workstations could also be used. All the experiments were conducted at room temperature. X-ray Photoelectron Spectroscopy (XPS) was performed by a PHI Versaprobe 5000 Scanning X-ray Photoelectron Spectrometer.

### 2.2. Biosensor Fabrication

This estrogen biosensor was based on a platform that was designed and manufactured. This platform biosensor had been successfully used in the detection of other biomarkers, such as lysyl oxidase like-2, LOXL2, of metastasis of breast cancer [31], hemoglobin A1c, HbA1c, of diabetes [32,33] and T-Tau of neuro-degenerative disorder [33,34], and the details of the fabrication of the biosensor were given elsewhere [31,32,33,34]. This biosensor used a three-electrode configuration. Both working and counter electrodes were thin gold film of 50 nm in thickness. The thin gold film was deposited using roll-to-roll sputtering technique. This roll-to-roll process was an established industrial process in which each sensor was estimated to cost less than US$2 to manufacture. Hence, the process was very cost-effective and the gold electrode elements were very uniform and reproducible, which were very practical and unique for single-use, in vitro or in situ applications. The overall dimensions of an individual biosensor were 33.0 × 8.0 mm^2^. The working electrode area was 1.54 mm^2^, accommodating 10–15 µL of liquid test sample. The employment of known micro-fabrication processes, such as sputtering physical vapor deposition, laser ablation and thick-film printing techniques, resulted in producing high-reproducible and low-cost, single-use disposable biosensors. As mentioned, a more detailed explanation of the electrode fabrication process can be found elsewhere [31,32,33,34].

### 2.3. Chemical Modification of the Biosensor

#### 2.3.1. Pretreatment of Gold Electrode (AuE)

As reported previously [33], a pretreatment procedure based on those described by others [35,36] was applied to the gold electrode, prior to the MPA-SAM deposition. This three-step pretreatment procedure resulted in a significant decrease in electrode charge transfer resistance, enhancing the reproducibility of the biosensor. A row of five or seven biosensors was immersed in a 2 M KOH solution for 15 min. After rinsing with copious amounts of deionized water, the biosensors were placed in a 0.05 M H_2_SO_4_ solution (95.0 to 98.0 w/w %) for another 10 min. DI water was then used to rinse the biosensor prototypes. The biosensors were then placed in a 0.05 M HNO_3_ solution (70 w/w %) for another 10 min. The biosensors were rinsed one more time with DI water and dried gently in a steam of nitrogen. The purpose of this pretreatment of the biosensor was to ensure the reproducibility of the biosensor, and the EIS study confirmed that this chemical pretreatment step was very effective. K_3_Fe(CN)_6_/K_4_Fe(CN)_6_ with 5 mM in each component was prepared in 0.1 M KCl for the EIS study. Concentrations of acids and base solutions used in this pretreatment procedure were optimized to be effective while maintaining the integrity of the thin gold film working and counter electrodes and the Ag/AgCl reference electrode, as well as the overall structure of the biosensor. The effectiveness of the pretreatment procedure was assessed using EIS and the results were excellent [33,34]. 

#### 2.3.2. Chemical Immobilization Steps on the Gold Electrode (AuE)

In this step, a thiol group was applied in order to provide a linkage between the anti-estrogen receptor and the gold electrode surface. Self-assembled monolayers of 3-Mercaptopropionic acid (MPA) were used for this purpose. MPA molecule consisted of a thiol functional group at one end, which provided an excellent affinity to gold, and a carboxylic group at another end, which was suitable for bonding covalently to proteins through peptide bond after an activation procedure. Thiol modification of gold electrode surface for protein immobilization was a well-acknowledged technique [33,34,37,38,39]. Typically, 4–8 biosensors were prepared in this immobilization step as a batch for this study. The biosensors were immersed in 50 mM solution of MPA in ethanol for 24 h in the dark, rinsed with DI water and dried in a steam of N_2_. The carboxylic groups on the other end of the MPA-modified AuEs were then functionalized by incubating in 0.1 M PBS (pH = 7.4) containing 0.25 M EDC and 0.05 M NHS for 5 h. Activated AuEs were then rinsed by 0.1 M PBS and dried by N_2_ flow; 20 µL of 45 µg/mL anti-estrogen receptor was casted on the sensing area of each AuE and left to dry overnight at 4 °C. Antibody immobilized biosensors were rinsed with 0.1 M PBS and immersed in 0.5 mM bovine serum albumin (BSA) in 0.1 M PBS solution for 2 h, preventing non-specific bonding. The biosensors were then rinsed with 0.1 M PBS again, dried under a steam of N_2_ and stored at 4 °C.

### 2.4. Characterization of the Biosensor

Prior to actual application, the characterization of the prepared biosensor was necessary to ensure that the biosensors were properly modified as designed. This investigation involved (1) the electrochemical analysis of bare, MPA-SAM-modified and antibody-attached biosensors; and (2) the degree of completeness in covering the biosensor in the chemical immobilization process.

In the electrochemical analysis of the biosensor at different stages of the modification, a solution of K_3_Fe(CN)_6_ and K_4_Fe(CN)_6_, with 5 mM in each component, was prepared in 0.1 M PBS and used as the redox coupled probe for DPV and EIS tests. In DPV measurement, it was anticipated that the bare biosensor would have the highest current output. Subsequently, the MPA-SAM- and antibody-modified biosensors would have lower current output indicating that the modification steps were successful. This observation was identical to that obtained in other biomarker detection of the platform biosensor technology [33,34], and the data will not be included here. EIS tests were performed in the Frequency range of 10^−2^ to 10^4^ Hz with 5 mV voltage amplitude. Randles equivalent circuit models were used to fit the Nyquist plots of EIS using EC-lab standard software.

X-ray photoelectron spectroscopy was used in the assessment of the degree of completeness in covering biosensors through the chemical process. Similar to our study of this platform biosensor [33], XPS high-resolution spectra of C(1s) and S(2p) obtained for MPA-SAM-modified AuE at the take-off angles of 10°, 50° and 90° were examined. The experimental results confirmed that there were fewer numbers of carboxylic groups near the surface. This observation confirmed the upward orientation of MPA-SAM carboxylic groups in this MPA-SAM arrangement as identical to the data given in previous study of this platform biosensor [33,34].

## 3. Results and Discussion

### 3.1. Preparation of Different Concentrations of 17 β-Estradiol Testing Solution

**Figure 1:**
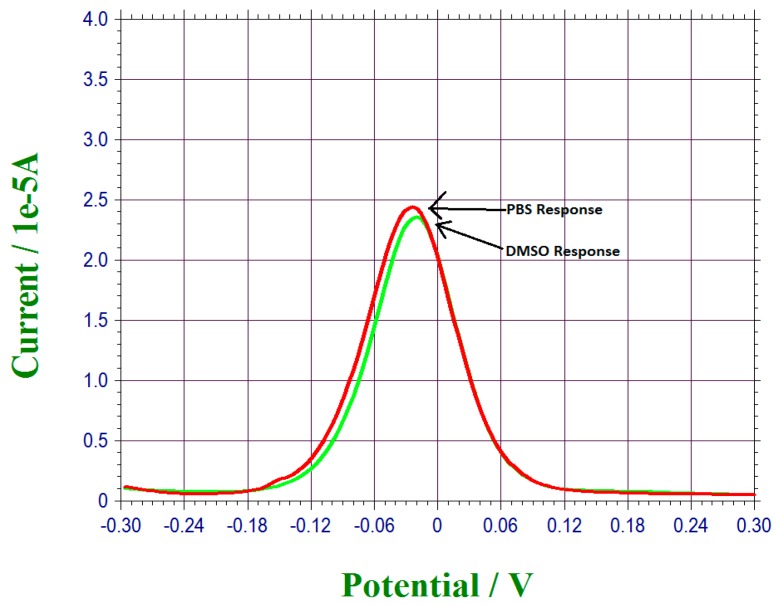
DPV measurements of DMSO and PBS solution indicating that DMSO as a solvent for 17 β-estradiol will not contribute to any current output in DPV measurement as compared to that in PBS. DPV, differential pulse voltammetry; DMSO, Dimethyl sulfoxide; PBS, Phosphate buffer saline.

17 β-estradiol had a limited solubility in PBS, distilled water and other aqueous solutions. However, it can be dissolved completely in dimethyl sulfoxide (DMSO) [39]. Consequently, 17 β-estradiol was first dissolved in DMSO in order to prepare different concentrations of 17 β-estradiol for testing. Thus, any potential effect of DMSO in the electrochemical measurement must first be assessed. Experimentally, differential pulse voltammetry (DPV) of our biosensor in pure DMSO and in 0.1 M PBS solution were carried out and the results were compared. Figure 1 shows the DPV measurement in DMSO and PBS solution. The nearly identical current outputs in the DPV measurements, as shown in Figure 1, suggest that DMSO did not contribute to any electrochemical effect as compared to PBS in DPV measurement using this biosensor. Similarly, 17 β-estradiol dissolved in DMSO would not contribute to any electrochemical current in tap water and simulated urine test solutions.

### 3.2. 17 β-Estradiol Detection in 0.1 M PBS

The detection of 17 β-estradiol was based on the effect on the redox reaction, K_3_Fe(CN)_6_/K_4_Fe(CN)_6_ affecting by the interaction between 17 β-estradiol and its α anti-estrogen receptor (17, 25–30). The anti-estrogen receptor used in this study was α-antibody of estrogen. Differential pulse voltammetry (DPV) was used in this study. The reaction between 17 β-estradiol and α-antibody of estrogen was irreversible. Thus, the DPV measurement of this interaction measured only the Faradic current, which was a diffusional control reaction influenced by the concentration of the 17 β-estradiol. Furthermore, DPV waves were affected by parameters, including the electrode reaction rate constant, transfer coefficient, waveform parameters. Consequently, the minor potential shift of the DPV waveform was due to these factors [33,34,40].

The MPA and EDC + NHS-modified biosensor was then attached with α-antibody of estrogen. The concentration of α-antibody of estrogen used was 45 µg/mL. The concentration of the 17 β-estradiol antigen used in this study was in the range of 2.25–2250 pg/mL. Preparation of the 17 β-estradiol in the PBS required a carefully developed procedure; 0.02 g of 17 β-estradiol antigen was placed in 1 mL of DMSO, 10 µL of this 17 β-estradiol-DMSO mixture was then added to 30 mL of PBS, And 10 µL of this solution was then added to 30 mL of PBS, resulting in a 2250 pg/mL of 17 β-estradiol in PBS. One mL of this 2250 pg/mL solution was then added into 9 mL PBS, resulting in a 225 pg/mL 17 β-estradiol in PBS. Concentrations of 17 β-estradiol in PBS of 22.5 pg/mL and 2.25 pg/mL were prepared in a similar manner, sequentially. The biosensor was prepared with the α-receptor antibody as described in Section 2.3.2, then 20 µL of the 17 β-estradiol antigen in PBS was placed on top of the biosensor. The biosensor was then incubated at room temperature for three hours and then rinsed with 0.1 M PBS and dried with N_2_ gas. A redox solution, K_3_Fe(CN)_6_/K_4_Fe(CN)_6_ was prepared using 5 mM equally of K_3_Fe(CN)_6_ and K_4_Fe(CN)_6_ in 0.1 M PBS solution; 20 µL of this redox solution was then added on top of the biosensor, and DPV measurement was then made. 

**Figure 2:**
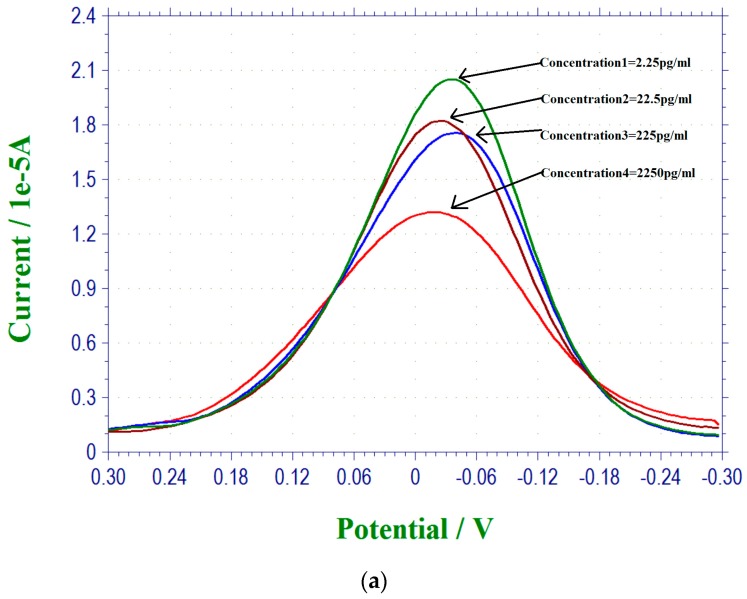

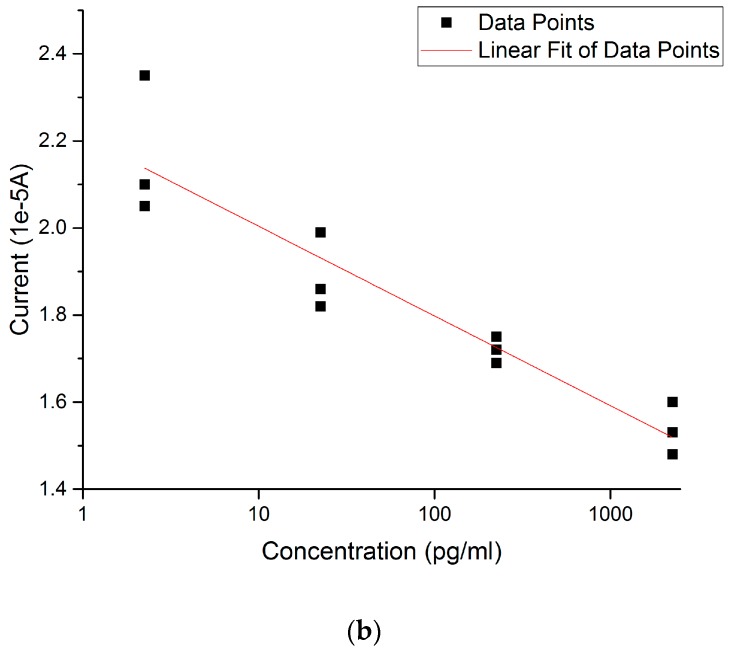
(**a**) DPV measurement of 17 β-estradiol over the concentration range of 2.25–2250 pg/mL in 0.1 M PBS solution; (**b**) Calibration curve of the DPV outputs and 17 β-estradiol concentration in 0.1 M PBS solution. Anti-estrogen receptor concentration is 45 µg/mL.

Figure 2a shows the DPV measurements of 17 β-estradiol antigens in 0.1 M PBS solution and Figure 2b shows the calibration curve based on the DPV measurements in Figure 2a. All the measurements from Figure 2 were conducted by the single-use disposable biosensor.

### 3.3. 17 β-Estradiol Detection in Tap Water from Cleveland, Ohio Regional Water District

**Figure 3:**
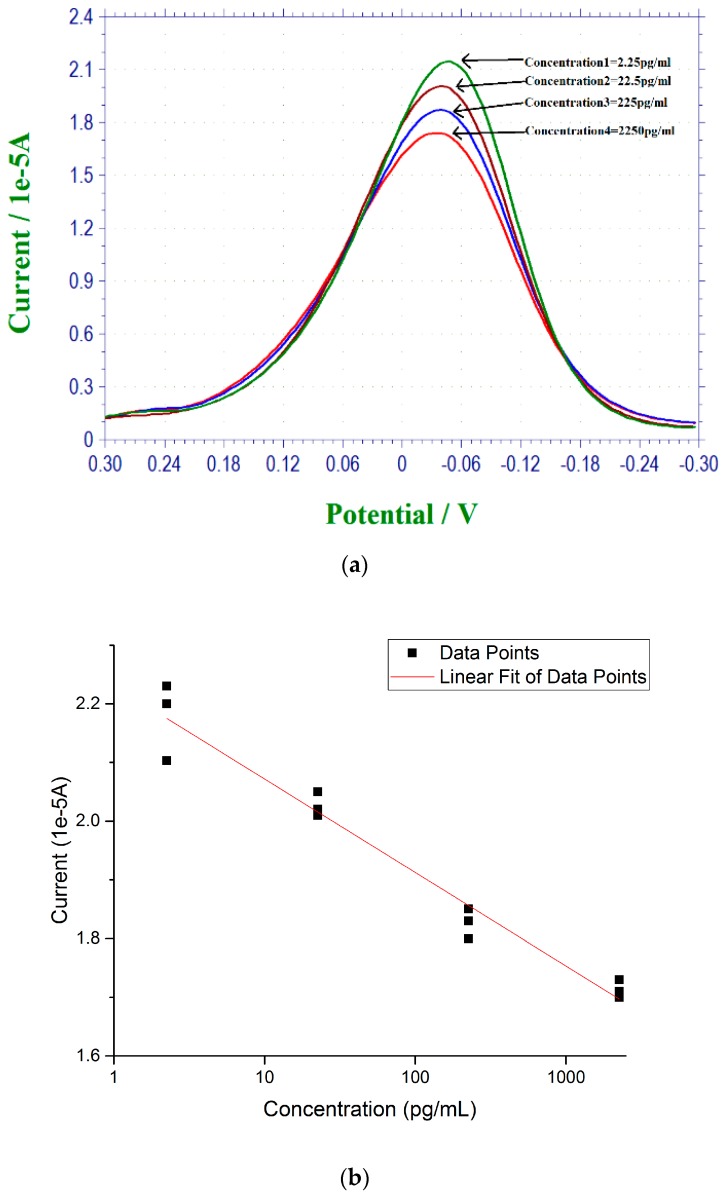
(**a**) DPV measurements of 17 β-estradiol antigen in the tap water samples; (**b**) The calibration curve of the 17 β-estradiol antigen detection in tap water samples based on the DPV measurement from Figure 3a with *n* = 3.

Estrogen pollution is an environmental concern, and the goal of this study includes the development of a simple in situ biosensor for 17 β-estradiol detection in regular water systems. Therefore, the regular tap water from the Cleveland regional water district was used as a test medium. 17 β-estradiol antigen was used to spike the tap water test sample providing the range of the 17 β-estradiol antigen for detection in a typical tap water sample. The range of the concentration of 17 β-estradiol antigen in the tap water sample was 2.25–2250 pg/mL, which was prepared in the same manner as in the PBS solution. DPV measurements of the 17 β-estradiol antigen were similar to the measurements of 17 β-estradiol antigen in PBS. Figure 3a shows the DPV measurement of the current outputs of the biosensor covering the 17 β-estradiol antigen concentration range of 2.25–2250 pg/mL in tap water from the Cleveland regional water district. Figure 3b is the calibration curve based on the DPV measurements from Figure 3a with *n* = 3.

### 3.4. 17 β-Estradiol Detection in Simulated Urine Test Sample

**Figure 4:**
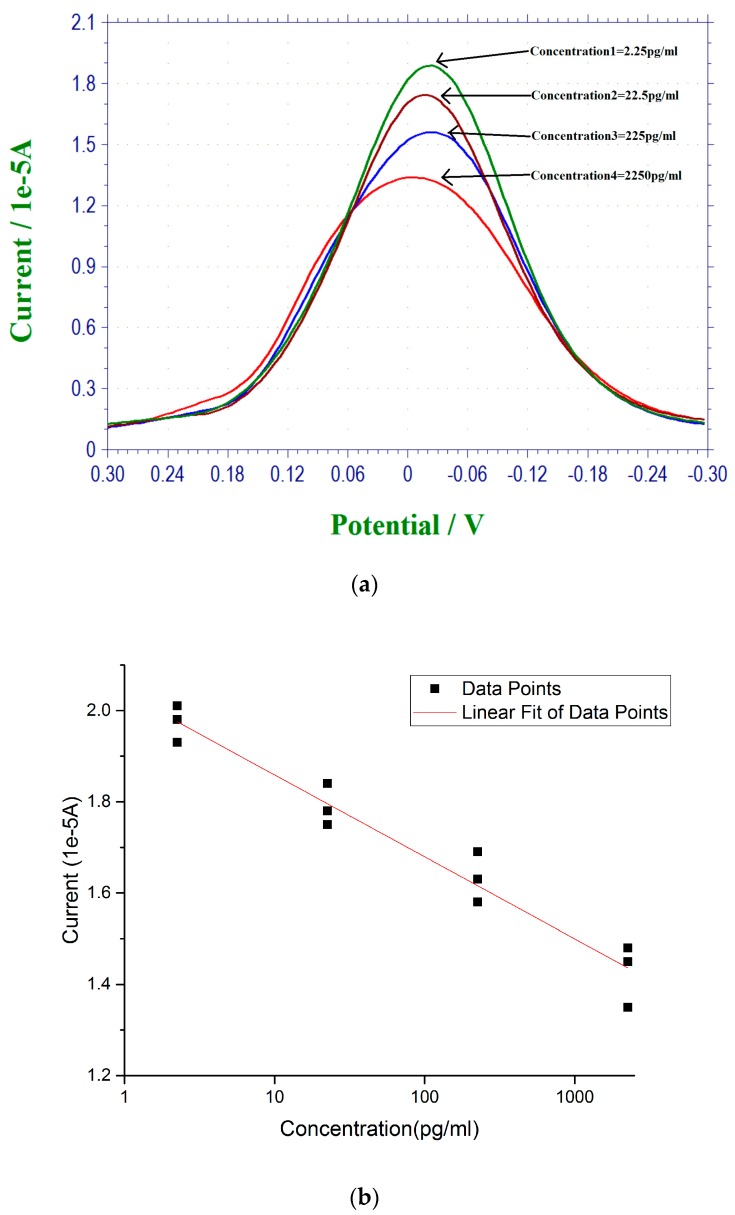
(**a**) DPV measurement of 17 β-estradiol antigen over the concentration range of 2.25 to 2250 pg/mL in simulated urine; (**b**) Calibration curve of the DPV outputs and 17 β-estradiol concentration in simulated urine. Anti-estrogen receptor concentration is 45 µg/mL.

Estrogen is directly related to the health of humans, particularly women. While the health implication of estrogen to woman is beyond the scope of this study, the development of a single-use in vitro biosensor for 17 β-estradiol antigen detection applicable to health care was one of the main focuses of this study. Specifically, this biosensor should be simple to use and would not require expensive instruments or skillful operators. In this phase of the study, simulated urine, normal (Cat. #695955) was purchased from the Carolina Biological Supply Co. (Burlington, NC, USA) and used. Urine sample is a non-invasive clinical procedure and it is very practical for in vitro testing. Figure 4a shows the 17 β-estradiol antigen measurements in the simulated urine samples using DPV measurements. The 17 β-estradiol antigen concentration range was 2.25–2250 pg/mL. Figure 4b is the calibration curve based on the DPV measurement from Figure 4a with *n* = 3.

### 3.5. Interference Study of This 17 β-Estradiol Biosensor

**Figure 5:**
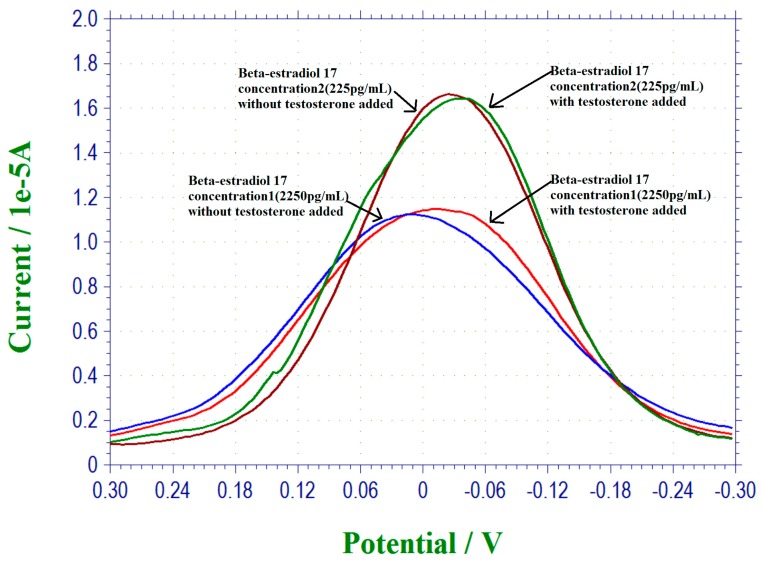
DPV measurements of estradiol 17 at the concentration of 225 pg/mL and 2250 pg/mL in the presence and absence of equal quantity of testosterone.

The selectivity and specificity of a biosensor is important in any meaningful development of a biosensor. This suggests that the biosensor should not be subject to interference by other hormones or biomarkers while in use. In this study, we chose testosterone as a potential interference in the detection of 17 β-estradiol study. The justification of selecting testosterone in this investigation was based on the similar chemical structure between 17 β-estradiol and testosterone, C_18_H_24_O_2_ and C_19_H_28_O_2_, respectively [39,41]. Also, the molecular weights between 17 β-estradiol, 272.388 g/mol and testosterone, 288.431 g/mol [41,42] were close and were useful in this interference study. Testosterone was a controlled substance, and special permission was needed to obtain this chemical. In this phase of the study, four different 17 β-estradiol antigen concentrations were used, namely, 2.25 pg/mL, 22.5 pg/mL, 225 pg/mL and 2250 pg/mL. At each 17 β-estradiol antigen concentration, an equal quantity of testosterone was then added into the test medium. PBS was used as the test medium. The current outputs of the DPV measurement of the biosensor in the presence and absence of the testosterone were nearly the same, indicating that testosterone will not interfere with this 17 β-estradiol biosensor, and suggesting that the selectivity of this biosensor based on the bio-recognition mechanism was very good and unique. Figure 5 shows the selected results of this interference study. Only the interference studies at 17 β-estradiol concentrations of 225 pg/mL and 2250 pg/mL are shown in Figure 5. The current outputs of the DPV measurements in the presence and the absence of testosterone are identical. The biosensor was used only once and was disposable. The performance as shown in Figure 5 not only demonstrates the good selectivity (non-interference) of this biosensor, but also the repeatability of this 17 β-estradiol biosensor.

## 4. Conclusions

A single-use, disposable in vitro or in situ 17 β-estradiol biosensor was designed, fabricated and produced. The design, modification and manufacturing of the biosensor was based on a platform technology which can be produced cost-effectively. It was simple to operate and did not require expensive instrument or skillful operators. The detection mechanism was based on the influence on the redox coupling reaction of K_3_Fe(CN)_6_/K_4_Fe(CN)_6_ affecting by the 17 β-estradiol antigen and its µ-antibody receptor. Evaluation of this 17 β-estradiol biosensor was carried out in PBS, tap water of the Cleveland regional water district, and simulated urine, with a concentration range of 2.25 pg/mL to 2250 pg/mL 17 β-estradiol antigen. Thus, this biosensor was applicable in estrogen pollution and health care estrogen detection systems. Chemical modification and functionalization of the immobilization of the µ-antibody receptor to the gold film-based biosensor were described. Interference study using testosterone, which had a similar chemical structure and molecular weight to 17 β-estradiol, was carried out and the results indicated that testosterone did not interfere with this 17 β-estradiol biosensor. Differential pulse voltammetry (DPV) was employed as the transduction mechanism for this biosensor. This research suggested that a cost-effective, single-use, disposable in vitro or in situ 17 β-estradiol biosensor could be used effectively for estrogen pollution in the environment, as well as for health care estrogen detection systems. This biosensor was produced cost-effectively, and is suitable for single-use, disposable in vitro or in situ applications.
